# ARL2 overexpression inhibits glioma proliferation and tumorigenicity via down-regulating AXL

**DOI:** 10.1186/s12885-018-4517-0

**Published:** 2018-05-29

**Authors:** Yulin Wang, Gefei Guan, Wen Cheng, Yang Jiang, Fengping Shan, Anhua Wu, Peng Cheng, Zongze Guo

**Affiliations:** 1 Department of Neurosurgery, The First Hospital of China Medical University, 155 Nanjingbei Street, Heping, Shenyang, Liaoning 110001 People’s Republic of China; 2 Department of Immunology, School of Basic Medical Science, China Medical University, Shenyang, 110122 Liaoning China

**Keywords:** ARL2, Glioma, AXL, Tumorgenecity, Brain cancer

## Abstract

**Background:**

Glioma is the most common primary brain tumor in adults with a poor prognosis. As a member of ARF subfamily GTPase, ARL2 plays a key role in regulating the dynamics of microtubules and mitochondrial functions. Recently, ARL2 has been identified as a prognostic and therapeutic target in a variety range of malignant tumors. However, the biological functional role of ARL2 in glioma still remains unknown. The aim of this study was to explore the expression and functional role of ARL2 in glioma.

**Methods:**

In this study, we investigated the expression of ARL2 in glioma samples by using RT-PCR, immunohistochemistry and western blot. The correlation between ARL2 expression and the outcomes of glioma patients was evaluated with survival data from TCGA, CGGA and Rembrandt dataset. Lentiviral technique was used for ARL2 overexpression in U87 and U251 cells. CCK8 assay, colony formation assay, wound healing test, transwell invasion assay and in vivo subcutaneous xenograft model were performed to investigated the biological functions of ARL2.

**Results:**

ARL2 expression was down-regulated in glioma, and was inversely associated with poor prognosis in glioma patients. Furthermore, exogenous ARL2 overexpression attenuated the growth and colony-formation abilities of glioma cells, as well as their migration and invasive capabilities. Moreover, elevated expression of ARL2 inhibited in vivo tumorigenicity of glioma cells. Mechanistically, ARL2 regulated AXL expression, which was known as an important functional regulator of proliferation and tumorigenicity in glioma cells.

**Conclusion:**

Our study suggests that ARL2 inhibits the proliferation, migration and tumorigenicity of glioma cells by regulating the expression of AXL and may conduct as a new prognostic and therapeutic target for glioma.

**Electronic supplementary material:**

The online version of this article (10.1186/s12885-018-4517-0) contains supplementary material, which is available to authorized users.

## Background

Glioma is the most common primary brain tumor in adults [1]. Although a standard treatment including extensive surgical resection followed by radiation and temozolomide chemotherapy has been adopted, the outcomes for glioma patients are still poor [1]. Median survival of glioblastoma multiforme (GBM), the most common and aggressive form of glioma, is 14–15 months and median progression-free survival (FPS) is approximately 6 months [1, 2]. Due to this dismal situation, great efforts have been made to find out effective approaches to halt the progression of this aggressive cancer. Besides this, recent studies have showed a tremendous understanding of the genetic and molecular mechanisms of glioma, leading to a renewed understanding about potential new therapeutic strategies, including oncogenic signal transduction inhibition/targeted therapy, anti-angiogenesis treatment, therapy targeting glioma stem cells, and immunotherapy [3].

Small G-proteins also known as the Ras superfamily structurally classified into 5 families: Ras, Rho, Rab, Sar/Arf, and Ran, which are involved in multiple cell signaling pathways and various cellular functions, including differentiation, proliferation, vesicle transport, nuclear assembly, and regulation of the cytoskeleton [4, 5]. Recent studies have identified Ras mutations in some human carcinomas [6–8]. It has been reported that activating mutations of KRAS-4B, within the mutated Ras family, occurs in approximately 21% of all human cancers, and accounts for approximately 90% of pancreatic cancers, 45% of colon cancers, and 30% of lung cancers, respectively [6]. The mutated forms of KRAS-4B not only activate their downstream signaling cascades, but also interact with each other and subsequently promote the proliferation of cancer cells and induce resistance to standard cancer therapies [6].

As a member of the ADP-ribosylation factor (ARF) subfamily, ADP ribosylation factor-like GTPase 2 (ARL2) is highly conserved and ubiquitously expressed in eukaryotes [9]. Previous studies show that ARL2 regulates microtubule dynamics through the interaction with tubulin-folding cofactor D (TBC-D), which is required for multiple mitochondrial functions including mitochondrial morphology, motility, asymmetric division, and maintenance of ATP levels [10–12]. Similarly, the trimer consisting of ARL2, tubulin-specific chaperone D and beta-tubulin is required for the maintenance of microtubule network [13–15]. In addition, ARL2 has been proved to be a fundamental regulator of farnesylated cargo and mitochondrial fusion [16, 17]. ARL2 is also involved in regulating nuclear retention of STAT3 with binder of ADP-ribosylation factor-like two (BART) [18–20]. Furthermore, ARL2 inhibition induces the apoptosis of neural progenitor cells derived from human embryonic stem cells [21]. However, the function role of ARL2 in cancer is still controversial. It has been reported that ARL2 expression level modifies cell morphology and influences mitotic and cytokinetic progression in breast cancer [22]. Recent study demonstrates that ARL2 expression is dramatically elevated in hepatocellular carcinoma and might be potentially utilizable as a prognostic marker [23]. Similarly, another study reports that ARL2 functions as an oncogene in cervical cancer [24]. Nevertheless, there is a study showing that breast tumor cells with increased ARL2 content present reduced aggressivity, *both* in vitro *and* in vivo [25]. Decreased ARL2 expression is associated with the regulation of p53 localization and results in a chemoresistant phenotype in breast cancer via a protein phosphatase 2A (PP2A) mediated mechanism [26]. Moreover, the pathophysiologic role of ARL2 in glioma remains unclear.

In this study, we investigated the expression and functional role of ARL2 in glioma. We firstly proved that decreased ARL2 expression level was clinically correlated to the higher grades and poorer outcomes of glioma patients. Secondly, we found that ARL2 overexpression attenuated the proliferation, clone formation, migration, invasive and tumorigenic capabilities of glioma cells by regulating the expression of receptor tyrosine kinase AXL.

## Methods

### Patients and samples

Twenty-three patient samples were collected at the First Hospital of China Medical University from February to June in 2016, including 20 glioma samples (grade II, 3 cases; grade III, 9 cases; grade IV, 8 cases) and 3 non-tumor brain tissue samples (from partial lobectomy in patients with epilepsy). Nine glioma tissues (grade II-IV, 3 cases for each grade) and 3 non-tumor brain tissue samples were used for qPCR and western blot. To further confirm the data of qPCR and western blot, IHC staining were performed with these 12 samples and other 11 glioma samples (grade III 6 cases and grade IV 5 cases). All glioma patients underwent surgical resection and the histological diagnosis was verified by 2 neuropathologists according to 2016 World Health Organization (WHO) guidelines. All of the samples used for this study were primary tumor samples, except 3 recurrent samples used for IHC staining. This study was approved by the Medical Ethics Committee of the First Hospital of China Medical University, and written informed consent was obtained from each patient. The clinical characteristics of 20 glioma patients were listed in Table 1.

**Table 1:** The clinical characteristics of 20 glioma patients

Characteristics	Number of patients (*n* = 20)
Age(years)	
<50	8 (40%)
≥ 50	12 (60%)
Gender	
Male	12 (60%)
Female	8 (40%)
WHO grade	
II	3 (15%)
III	9 (45%)
IV	8 (40%)
Tumor size	
<4 cm	11 (55%)
≥ 4 cm	9 (45%)
Primary/Recurrent	
Primary	17 (85%)
Recurrent	3 (15%)
IDH1 state	
IDH1(−)	11 (55%)
IDH1(+)	9 (45%)

### Cell culture

U87-MG (catalogue number TCHu58) and U251 (catalogue number TCHu138) cell lines were obtained from the Chinese Academy of Sciences Cell Bank (Shanghai, China) and maintained in high-glucose Dulbecco’s Modified Eagle’s Medium (DMEM) (Hyclone, SH30022.01) supplemented with 10% fetal bovine serum (FBS, Hyclone, SV30087), 100 U/ml of penicillin, and 100 U/ml of streptomycin (Hyclone, SV30010) at 37 °C with 5% CO_2_.

### RNA isolation and quantitative RT-PCR (qPCR)

Total RNA was isolated from U87, U251 cells and 12 clinical samples using TRIzol reagent (Invitrogen), according to the manufacturer’s protocol. Total RNA was reversely transcribed into cDNA and used for PCR amplification. Real-time PCR were performed in thermal cycler (Roche LightCycler 480) using *TransStart*^®^ Top Green qPCR SuperMix Assays (Transgen Biotech, AQ131). PCR conditions were as follows: 1 cycle of 95 °C for 30s, followed by 40 cycles of a two-step cycling program (95 °C for 5 s; 60 °C for 30s). The mRNA expression was normalized to the expression of *GAPDH* mRNA and calculated by the 2^-ΔΔCt^ method. Specific primers for *ARL2*, *AXL* and *GAPDH* were: *ARL2* forward: GGGAGGACATCGACACCA and reverse: AGGACCGCAGGGACTTCT [27]; *AXL* forward: 5-GTTTGGAGCTGTGATGGA AGGC-3 and reverse: 5-CGCTTCACTCAGGAAATCCTCC-3 [28]; *GAPDH* forward: GAAGGTGAAGGTCGGAGTCA and reverse: TTGAGGTCAATGAAG GGGTC [29], respectively.

### Protein extraction and western blot analysis

Total proteins from tissue and cells were extracted by whole cell lysis buffer (Wanleibio) and quantified using the bicinchoninic acid (BCA) method. 30 μg of protein from each sample was electrophoresed by 12% SDS-PAGE and transferred to PVDF membranes (0.45 μm, Millipore). After being blocked with 5% skimmed milk or 5% BSA (used for phosphorylated protein), the PVDF membranes were incubated overnight at 4 °C with the primary antibody. Membranes were then washed three times with TBST (5 min each), and incubated with peroxidase-conjugated affinipure goat anti-rabbit (1:5000; Proteintech) or anti-mouse (1:10000; Proteintech) IgG at 37 °C for 1 h. Protein expression was visualized with a chemiluminescence ECL kit (Tanon, 5500). GAPDH served as a loading control, and band intensity was quantified using Image J software.

### Immunohistochemistry and immunocytochemistry

For immunohistochemistry, all samples were fixed in 10% neutral formalin and embedded in paraffin. Sections (4 μm thick) were cut from paraffin blocks and mounted on Poly-L-Lysine-coated glass slides. The sections were deparaffinized in xylene and rehydrated in gradient ethanol. Antigen retrieval was performed in 0.01 mol/L citrate buffer (pH 6.0) by microwave oven for 15 min at 95 °C. Endogenous peroxidase activity was blocked with 3% hydrogen peroxide for 10 min and the sections were incubated with normal goat serum to reduce nonspecific binding for 15 min. Sections were incubated with primary antibody in blocking solution at 4 °C overnight in a humidified chamber. After washing three times with PBS, sections were incubated with biotinylated goat anti-rabbit IgG (SP-9001, ZSGB-BIO) for 15 min at room temperature. After washing in PBS, 3, 3′-diaminobenzidine (DAB) was used for developing. Slides were counterstained with hematoxylin for 3 min. Then the sections were dehydrated and mounted with coverslips. German immunohistochemical score (GIS) was applied to evaluate the expression of ARL2 [30]. Percentage of positive cells was classified as 0 (negative), 1 (up to 10%), 2 (11–50%), 3 (51–80%), or 4 (> 80% positive cells), staining intensity was classified as 0 (no staining), 1 (weak), 2 (moderate), or 3 (strong). The final immunoreactive GIS were defined as the multiplication of both grading results (percentage of positive cells × staining intensity). The IHC expression value of ARL2 and related sample information were listed in Table 2.

**Table 2:** The ARL2 expression value and information of samples used for IHC

Sample tissues (23 cases in total)		Sample NO.	Age range (years)	GIS score	*P* value (vs. non-tumor)
Non-tumor tissue (3 cases)		N1	38–65	12	
		N2		12	
		N3		12	
Glioma tissues	Grade II (3 cases)	G0201	26–60	4	0.0074
		G0202		9	
		G0203		6	
	Grade III (9 cases)	G0301	32–70	2	0.0001
		G0302		2	
		G0303		1	
		G0304		3	
		G0305		8	
		G0306		2	
		G0307		1	
		G0308		0	
		G0309		0	
	Grade IV (8 cases)	G0401	37–79	4	0.0001
		G0402		2	
		G0403		0	
		G0404		1	
		G0405		2	
		G0406		4	
		G0407		4	
		G0408		1	

For immunocytochemistry, 5 × 10^3^ cells were seeded into confocal dish per well and incubated at 37 °C with 5% CO_2_ for 24 h. Then the cells were fixed with 4% paraformaldehyde and permeated with 0.3% Triton X-100 for 20 min. After blocking with 5% BSA for 1 h, primary antibody was added and incubated at 4 °C overnight. Following incubation with rhodamine(TRITC)-conjugated affinipure goat anti-rabbit IgG (Proteintech, SA00007–2) and DAPI (BOSTER, AR1176), the samples were detected using fluorescence microscope (OLYMPUS, BX53).

### ARL2 expression data mining in GEO dataset, TCGA and Rembrandt dataset

*ARL2* expression data of TCGA and Rembrandt dataset and the patients’ survival data of Rembrandt dataset were extracted from Project Betastasis (http://betastasis.com/). The patients’ survival data of TCGA were downloaded from GlioVis portal (http://gliovis.bioinfo.cnio.es). In addition, GEO datasets (GSE50161, Griesinger dataset; GSE4290, Sun dataset) were applied to analyze the expression level of ARL2 in glioma and normal brain [31, 32]. Gene Set Enrichment analysis (GSEA, www.broadinstitute.org/gsea/index.jsp) was applied to obtain the functional information on ARL2 as previously described [33, 34]. Moreover, the data from Chinese Glioma Genome Atlas (CGGA) were used to analyze ARL2 expression and the patients’ survival time [35, 36]. The relevant signaling pathways of ARL2 from KEGG and Reactome were analyzed by pathDIP (http://ophid.utoronto.ca/pathdip/).

### Lentivirus mediated ARL2 and AXL over-expression

Lentiviruses carrying overexpressing ARL2, AXL and control vectors were purchased from GeneChem (Shanghai, China). The lentivirus transduction was performed according to the protocol provided by the company. In brief, after the cells (10^5^ cells/well in 1 ml high-glucose DMEM medium supplemented with 10% FBS) were seeded in a 6-well plate for 24 h, 20 μl of lentivirus solution (10^7^ IU/mL) were added to each well and the cells were incubated at 37 °C with 5% CO_2_ for 12 h. The medium was replaced with fresh DMEM medium containing 10% FBS. After 48 h of transduction, the cells were selected with puromycin (10 μg/mL). Medium was changed every 3 days. Real-time PCR and western blot were performed to assess the transfected efficiency.

### In vitro cell proliferation assays

5 × 10^3^ cells in 100 μl medium were seeded into 96-well plates per well and incubated at 37 °C with 5% CO_2_ for 6 days. The cell proliferation was measured at day 0, 2, 4 and 6 by adding 10 μl CCK8 (DojinDo) into the wells and following 4 h incubation at 37 °C. Then OD values of each well were measured by microplate reader (BIO-RAD 15033) at the absorbance of 450 nm. Growth curves were plotted according to the OD value of each well.

### Colony formation assay

After transduced with ARL2, AXL overexpression or control lentiviruses for 72 h, the cells were collected and resuspended as single cells. Cells were seeded into the wells of a six-well plate and incubated at 37 °C with 5% CO_2_. After 2 weeks, the cells were washed with PBS twice and stained with crystal violet staining solution. The number of colonies (more than 50 cells) was counted under a microscope (Leica, 090–135.001).

### Wound healing test

The cells (5 × 10^5^ per well) were seeded into six-well plates. After 24 h, the cells overspread the bottom and were scratched by a 200 μl pipette tip. PBS was used to wash out cell debris and suspension cells. Fresh serum-free medium was added, and the cells incubated at 37 °C with 5% CO_2_ to allow the wound to heal. Photographs of the wound were taken at 0 and 24 h at the same position. The percentage of wound closure was measured according to previous reports [37, 38]. In brief, the wound areas were evaluated by image J software and the percentage of wound closure were calculated via the formula as follow: (original wound area - actual wound area)/area of the original wound × 100.

### Transwell invasion assay

Transwell chambers with a pore size of 8 μm filter membrane (Corning, 3422) were used to perform invasion assay. 100 μl Matrigel (Corning, 356,234) (diluted with serum-free DMEM by 1:8) was plated in transwell chamber, and preserved in an incubator. Four hours later, 200 μl of serum-free medium containing 10^5^ cells was added into top chambers of transwell inserts, and 750 μl DMEM containing 10% FBS was added into bottom chambers. The cells were incubated at 37 °C in 5% CO_2_ for 16 h. Matrigel and cells in top chambers were removed by cotton swab. After fixation with 4% paraformaldehyde, the cells traversing the membrane were stained with crystal violet staining solution and counted under five different high-power microscope fields per well. The experiment was performed in triplicate.

### In vivo subcutaneous tumor transplantation

All animal procedures were conformed to protocols approved by the Animal Care Committee of China Medical University. For xenograft subcutaneous transplantation, 6-week-old male immune-deficient nude mice (BALB/C-Null) were purchased from Beijing Vital River Laboratory Animal Technology Company. Mice were raised in laminar flow cabinets under specific pathogen free (SPF) conditions and were fed ad libitum. U87 cells (transduced with ARL2 overexpression or control vector) were injected into the back flanks of nude mice at a density of 10^7^ cells per 0.3 ml as previous described [39, 40]. The tumor size was measured using a Vernier caliper per 4 days, and the tumor volume was calculated using the formula: V = (length x width^2^) / 2 [41]. The mice were sacrificed at day 28 after implantation, and the tumors were weighed and photographed.

### Statistical analysis

Data are presented as mean ± SD. The number of replicates for each experiment is stated in the figure legend. Statistical differences between and among groups were determined by two tailed *t*-test or one-way analysis of variance (ANOVA) followed by Dunnett’s post-test, respectively. Statistical analysis was performed by Microsoft Excel 2013 and Graphpad Prism 6.0, unless mentioned otherwise in the figure legend. *P* < 0.05 was considered as statistically significant.

## Results

### ARL2 expression is decreased in glioma

**Fig. 1:**
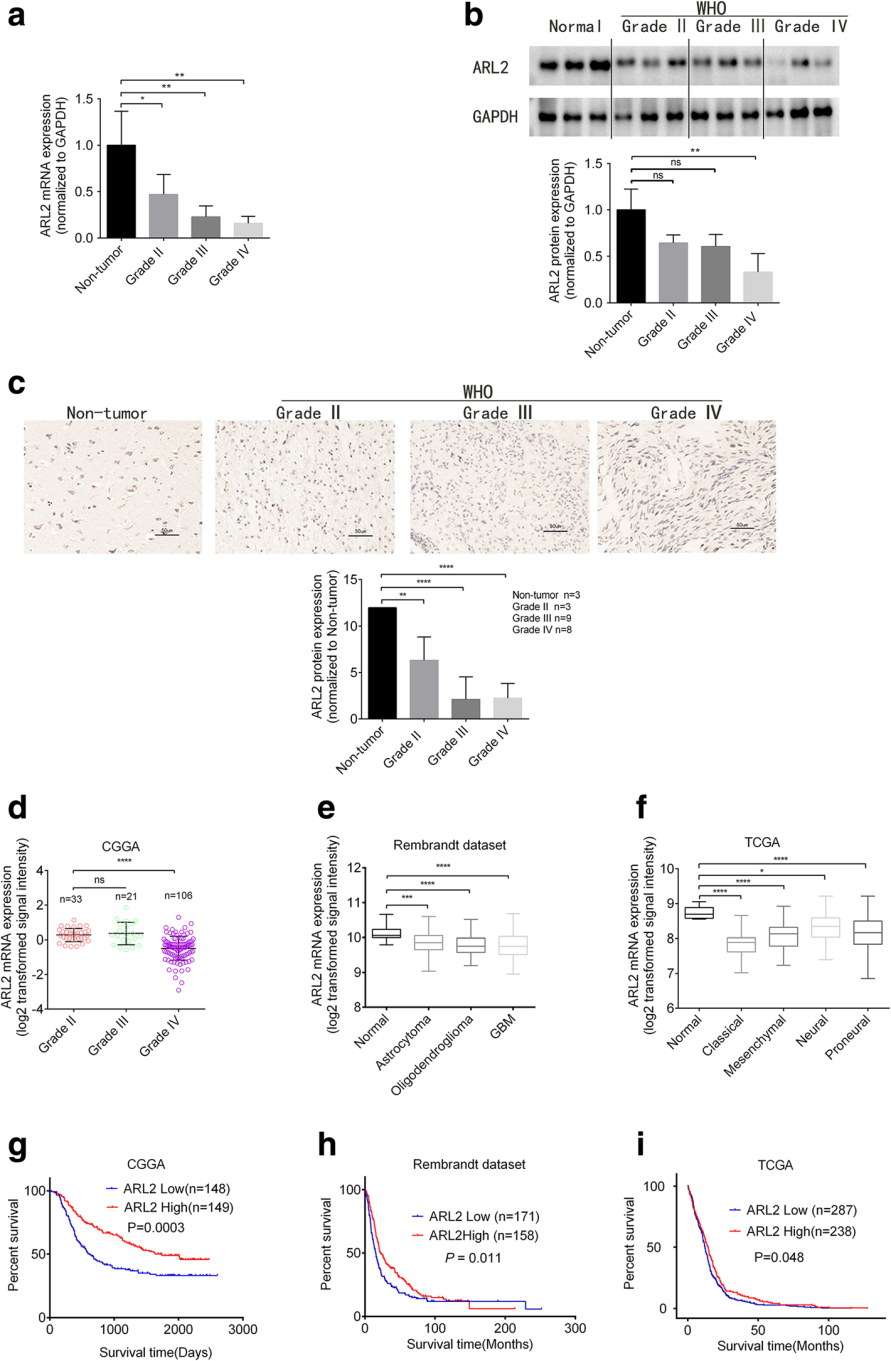
Decreased ARL2 expression is clinically relevant with poor prognosis of glioma patients. **a** qRT-PCR analyses of *ARL2* mRNA in WHO grade II-IV glioma and non-tumor samples (grade II, *n* = 3; grade III, *n* = 3; grade IV, *n* = 3, non-tumor *n* = 3) (non-tumor vs. grade II, *P* = 0.0489; non-tumor vs. grade III, *P* = 0.0075; non-tumor vs. grade IV, *P* = 0.0046; one-way ANOVA). **b** Western blot analyses of ARL2 protein in WHO grade II-IV glioma and non-tumor samples (grade II, *n* = 3; grade III, *n* = 3; grade IV, *n* = 3, non-tumor *n* = 3) (non-tumor vs. grade II, *P* = 0.0761; non-tumor vs. grade III, *P* = 0.0512; non-tumor vs. grade IV, *P* = 0.0033; one-way ANOVA). **c** Representative immunohistochemistry images and analyses of ARL2 protein in WHO grade II-IV glioma and non-tumor brain samples (grade II, *n* = 3; grade III, *n* = 9; grade IV, *n* = 8; non-tumor *n* = 3). Scale bar, 50 μm. (non-tumor vs. grade II, *P* = 0.0074; non-tumor vs. grade III, *P* < 0.0001; non-tumor vs. grade IV, *P* < 0.0001; one-way ANOVA). **d** Data from CGGA showed ARL2 mRNA expression decreased in grade IV compared to grade II (grade II, *n* = 33; grade III, *n* = 21; grade IV, *n* = 106) (grade II vs. grade IV, *P* < 0.0001; grade II vs. grade III, *P* = 0.8438, one way ANOVA). **e**, **f** Data from Rembrandt database (**e**, non-tumor, *n* = 28; astrocytoma, *n* = 148; oligodendroglioma, *n* = 67; GBM, *n* = 228) (non-tumor vs. Astrocytoma, *P* = 0.0007; non-tumor vs. oligodendroglioma, *P* < 0.0001; non-tumor vs. GBM, P < 0.0001; one-way ANOVA) and TCGA (F, normal, *n* = 11; classical, *n* = 54; mesenchymal, *n* = 58; neural, *n* = 33; proneural, *n* = 57) (normal vs. classical, *P* < 0.0001; normal vs. mesenchymal, *P* < 0.0001; normal vs. neural, *P* = 0.0188; normal vs. proneual,*P* < 0.0001 one-way ANOVA) revealed that ARL2 mRNA expression decreased in glioblastoma, compared with non-tumor brain tissues. **g**-**i** Data from CGGA (G, *P* = 0.0003, low, *n* = 148; high, *n* = 147), Rembrandt database (**h**, low, *n* = 171; high, *n* = 158) and TCGA (**i**, low, *n* = 287; high, *n* = 238) indicated ARL2 was opposite relevant to the poor prognosis of glioma patients

The expression of ARL family members in TCGA were studied through GlioVis, and ARL2 was significantly differentially expressed between glioma and non-tumor samples (Additional file 1: Figure S1). To further investigate ARL2 expression in glioma, we assessed mRNA and protein levels in a series of clinical glioma specimens and cell lines. Twelve human clinical specimens were collected including 9 glioma tissues (grade II-IV, 3 cases separately) and 3 non-tumor brain tissue samples. Quantitative PCR was performed on these specimens. The result showed that ARL2 mRNA levels decreased with the increase in grade of tumor tissues (Fig. 1a, *P* < 0.01), as well as U87 and U251 glioma cells (Additional file 2: Figure S2A, *P* < 0.0001). Similarly, ARL2 protein expression levels were down-regulated in grade IV glioma samples (Fig. 1b, *P* < 0.05), as well as U251 and U87 cells than non-tumor samples (Additional file 2: Figure S2B, *P* < 0.001). We then examined ARL2 expression in 20 gliomas tissue samples (grade II, 3 cases; grade III, 9 cases; grade IV, 8 cases) and 3 non-tumor brain tissue samples by immunohistochemistry. The data showed that ARL2 protein expression was reduced in high grade glioma samples (Fig. 1c, grade III, *P* < 0.01; grade IV, *P* < 0.0001). We also collected ARL2 expression data from CGGA, Rembrandt database and TCGA. The results confirmed that ARL2 expression level significantly decreased in GBM (grade IV) (CGGA, Fig. 1d, *P* < 0.0001; Rembrandt database, Fig. 1e, *P* < 0.0001). In TCGA, a decreased ARL2 expression could be observed in all subtypes (Proneural, Mesenchymal, Neural and Classical) of GBM, compared to non-tumor samples (Fig. 1f, *P* < 0.05). Data from Sun (Additional file 2: Figure S2C, *P* < 0.001) and Griesinger dataset (Additional file 2: Figure S2D, *P* < 0.05) also demonstrated the consistent results. Taken together, these results demonstrated that ARL2 expression significantly decreased in glioma.

### ARL2 expression is clinical relevant with the poor prognosis of glioma patients

Recently, ARL2 has been identified as a potential prognosis marker for hepatocellular carcinoma [23]. To examine whether ARL2 expression is associated with glioma patient outcomes, we analyzed the data from CGGA, Rembrandt database, and TCGA to investigate the clinical relevance of ARL2. The data from CGGA showed decreased ARL2 expression was clinical relevant to the poor prognosis of glioma patients (Fig. 1g, *P* = 0.0003). Similar results were obtained from Rembrandt, and the patients with a higher ARL2 expression had a favorable survival (Fig. 1h, *P* = 0.011). Finally, we verified the result in TCGA. The consistent elevated expression of ARL2 was also associated with prolonged survival (Fig. 1i, *P* = 0.048).

### ARL2 attenuated the growth and colony-formation abilities of glioma cells

**Fig. 2:**
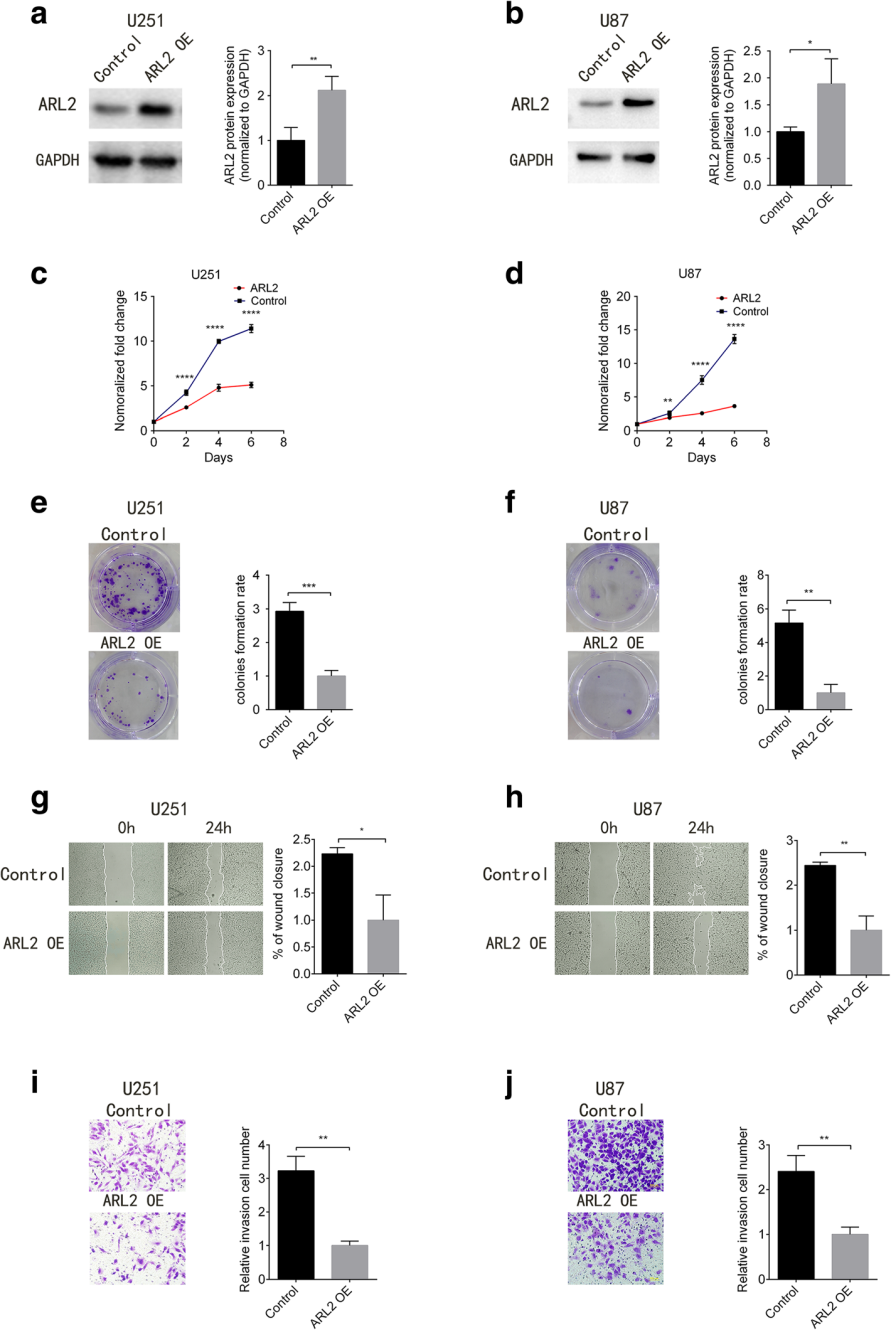
ARL2 regulated the growth, colony formation, migration and invasion capabilities of glioma cells. **a**, **b** The representative western blot images and analyses of ARL2 in U251 (**a**) and U87 (**b**) glioma cells transduced with lentiviral ARL2 vector or its control vector (U251, *P* = 0.0099; U87, *P* = 0.0316; *n* = 3; *t* test). **c**, **d** In vitro growth assay showed that ARL2 overexpression inhibited the proliferation of U251 (**c**) and U87 (**d**) (** *P* < 0.01; ****P < 0.0001; *n* = 5, *t* test). **e**, **f** Colony formation assay revealed that ARL2 overexpression reduces clone formation ability of U251 (**e**) and U87 (**f**) cell lines (500 cells/well; U251, *P* = 0.0004; U87, *P* = 0.0014; *n* = 3, *t* test). **g**, **h** Wound healing test showed that ARL2 overexpression decreased the migration abilities of U251 (**g**) and U87 (**h**) cells (U251, *P* = 0.0111; U87, *P* = 0.0015; *n* = 3, *t* test). **i**, **j** Transwell assay demonstrated that ARL2 overexpression inhibited the invasion capabilities of U251 (**i**) and U87 (**j**) cells. Scale bar, 100 μm. (*P* < 0.01, *n* = 3, with *t* test)

To investigate the physiological role of ARL2 in glioma, we first overexpressed ARL2 through transducing lentiviral ARL2 vector in glioma cell lines (U87 and U251), and then examined the effects on cell growth. qPCR and Western blotting assay showed that both of ARL2 mRNA and protein expression level were significantly elevated at 48 h after transduction (Fig. 2a, *P* = 0.0099, and b, *P* = 0.0316; Additional file 3: Figure S3A *P* < 0.0001, and S3B, *P* = 0.0075). As a result, ARL2 overexpression significantly suppressed the proliferation of U251 and U87 cells, compared with cells transduced with the control vector (Fig. 2c and d). Moreover, the colony formation assay was performed to examine the foci formation ability of these cells. As expected, foci formation abilities of U251 or U87 cells infected with lentiviral ARL2 overexpression vector were dramatically decreased in comparison with control cells (Fig. 2e and f). In addition, we measured the cell cycles in U251 cells transfected with or without ARL2 overexpression vector. The percentage of cells at G0/G1 phase in U251 cells with ARL2 overexpression was increased and the proportion at S and G2/M phase was decreased, oppositely (Additional file 3: Figure S3C, G0/G1 phase *P* = 0.0014, S phase *P* = 0.0049, and G2/M phase *P* = 0.0111). This data indicated ARL2 overexpression induced G0/G1 arrest in glioma cells and inhibited their proliferation. Taken together, these results indicated that ARL2 overexpression inhibited the growth and clonogenicity of glioma cells.

### ARL2 inhibited the migration and invasive capabilities of glioma cells.

Since microtubule network plays a crucial role in the regulation of cell migration and invasion, wounding healing test and Transwell invasion assay were performed to examine whether ARL2 overexpression inhibited the migration and invasion of glioma cells. As shown in Fig. 2g and h, glioma cells with ARL2 overexpression migrated significantly more slowly than control cells (Fig. 2g, *P* < 0.05, and H, *P* < 0.01). Similar results were obtained from Transwell assay, ARL2 overexpressed cells exhibited decreased invasive capabilities (Fig. 2i, *P* < 0.01, and j, *P* < 0.01). These data indicated that ARL2 diminished the migration and invasion abilities of glioma cells.

### ARL2 suppressed the tumorigenicity of glioma cells in vivo

**Fig. 3:**
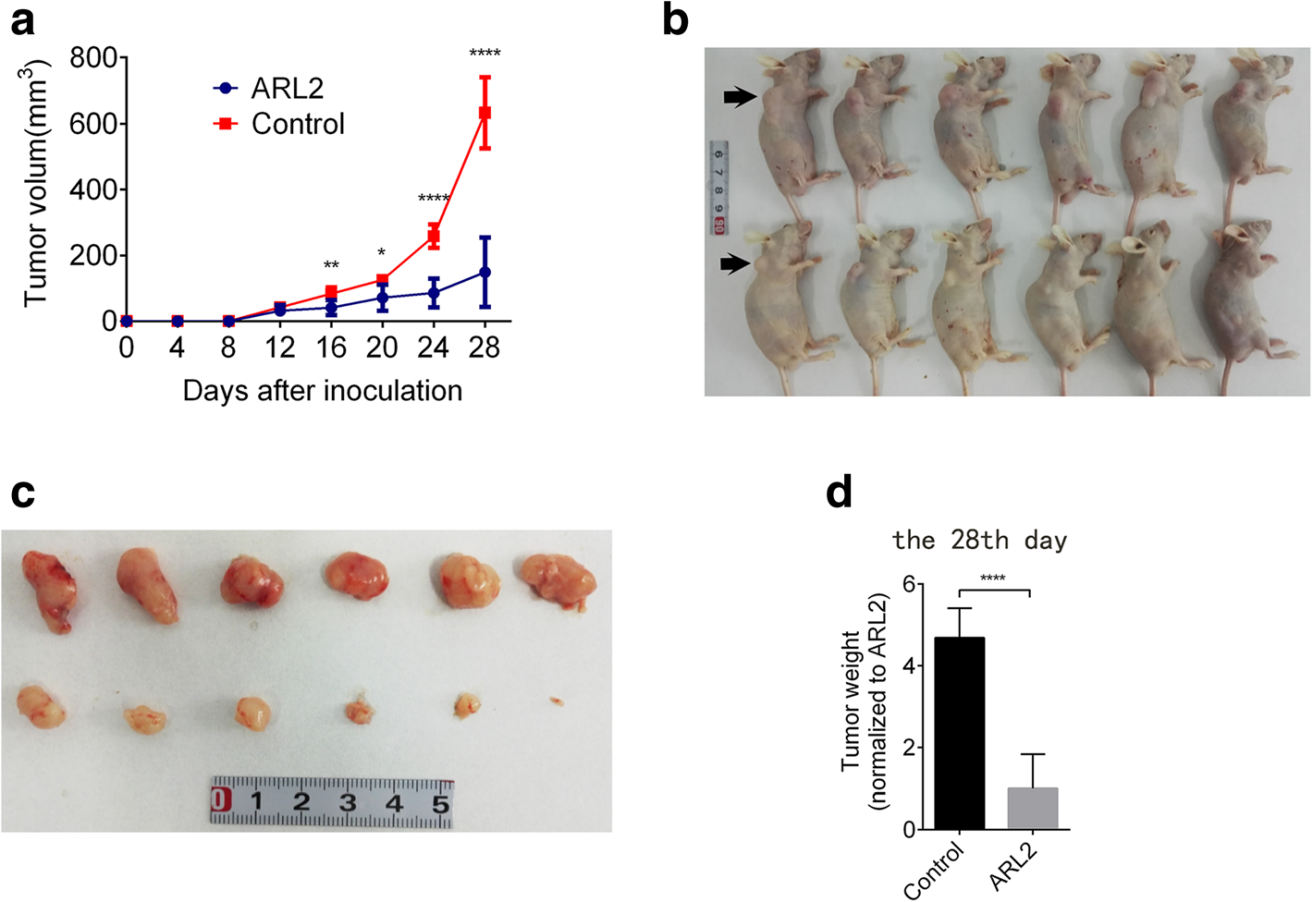
ARL2 inhibits in vivo tumor formation capability of glioma cells. **a** The size analysis of subcutaneous tumors measured every four days from nude mice transduce with ARL2 overexpression or control vector transduced U87 cells. **b**, **c** Images of mice (**b**) and subcutaneous (**c**) tumors at 28th day after subcutaneous transplantation of ARL2 overexpression or control vector transduced U87 cells. **d** The weight of tumor from nude mice at 28th day after transplantation of U87 cells transduced with ARL2 overexpression or control vector

To determine whether ARL2 is important to the tumorigenicity of glioma cells in vivo, we injected U87 cells infected with ARL2 overexpression vector or control vector into the flank regions of nude mice and measured tumor volumes every 4 days. The results demonstrated that the upregulation of ARL2 expression resulted in a reduction in subcutaneous growth of U87 glioma cells (Fig. 3a). Consistently, after 4 weeks of xenograft transplantation, although ARL2 overexpression didn’t alter the tumorigenesis, the mean volume and weight of subcutaneous tumors in ARL2 overexpression group were obviously smaller and lighter than the control group (Fig. 3b-d). Collectively, these results showed that ARL2 could suppress glioma tumorigenicity in vivo.

### ARL2 decreased AXL expression in glioma cells

**Fig. 4:**
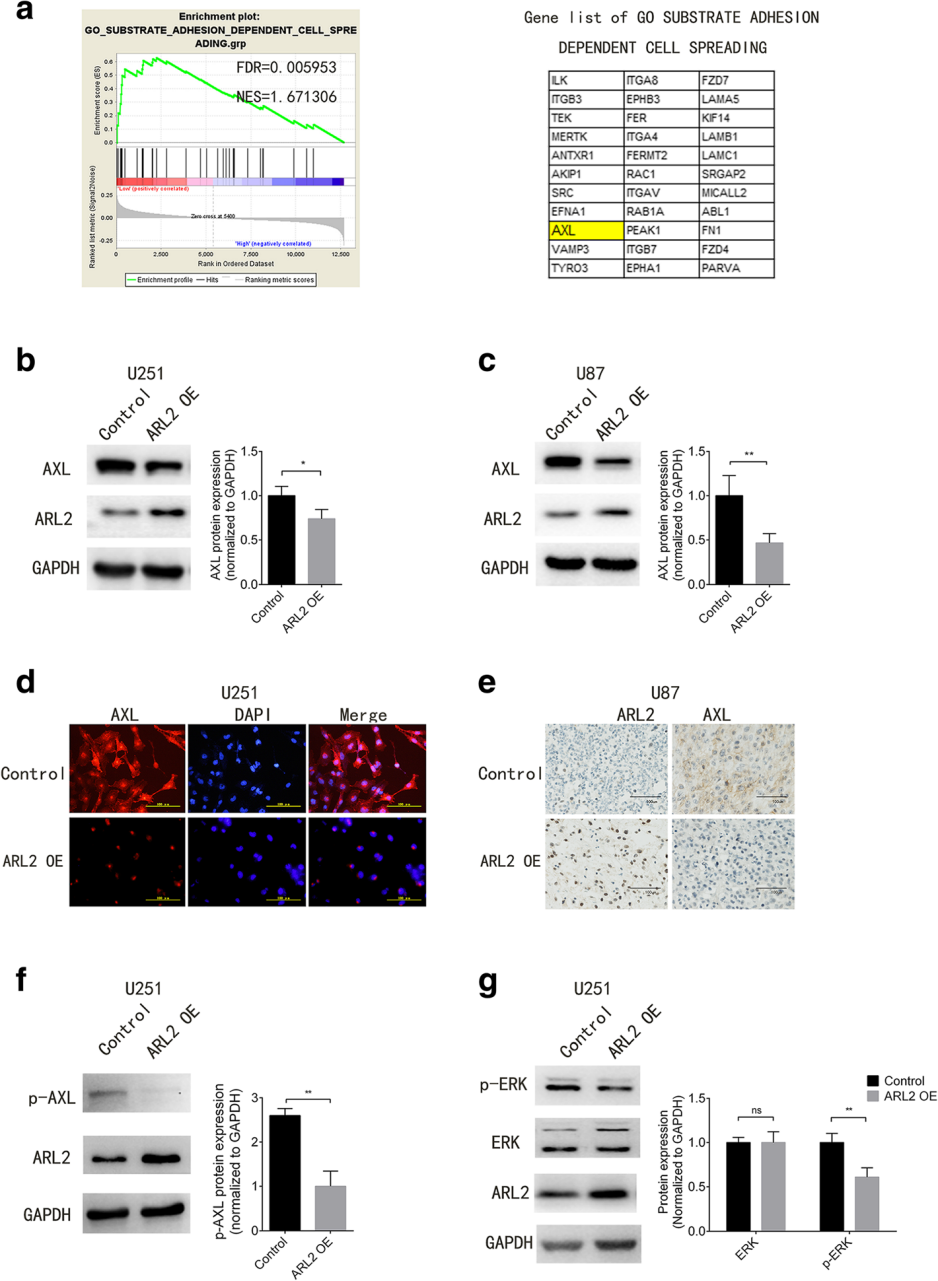
ARL2 decreases AXL expression in glioma cells. **a** GSEA analysis showed that the expression of ARL2 is associated with substrate adhesion dependent cell spreading signaling pathway (33 genes were enriched and AXL was involved, http://www.broadinstitute.org/gsea/index.jsp). The normalized enrichment scores (NES) and the *p* values are shown in the plot. **b**, **c** Representative images and analysis of western blot demonstrated that ARL2 overexpression decreased AXL protein expression in U251 (**b**) and U87 (**c**) cells. (U251, *P* = 0.038, *n* = 3; U87, *P* = 0.0053; *n* = 4, *t* test). **d** Representative images of immunocytochemistry showed that ARL2 overexpression decreased AXL expression. **e** The IHC staining of U87 xenograft confirmed that AXL expression decreased after ARL2 overexpression. Scale bar = 100 μm. **f** ARL2 overexpression decreased phospho-AXL protein expression in U251 cells (*P* = 0.0018, *n* = 3, *t* test). **g** ARL2 overexpression inhibited phospho-ERK expression (total ERK, *P* = 0.9975; phospho-ERK, *P* = 0.0019, *n* = 4, *t* test)

Due to the functional role of ARL2 in glioma, we investigated the downstream target of ARL2 via Gene Set Enrichment Analysis (GSEA). Adhesion dependent cell spreading signaling pathway were enriched, including 33 genes, such as ILK, ITGA8, and AXL (Fig. 4a). Furthermore, another two signaling pathway were enriched (epidermal growth factor and epidermal growth factor stimulus) (Additional file 4: Figure S4A and S4B). It has been reported that AXL is closely relevant to EGFR signaling pathway and mediates the resistance to EGFR inhibition in lung cancer and GBM [42–44]. We then explored the relationship among AXL and enriched genes in these two datasets separately via STRING (https://string-db.org/). The data also confirmed the close relationship between AXL and these enriched genes (Additional file 4: Figure S4C and S4D). Moreover, pathway analysis through pathDIP (http://ophid.utoronto.ca/pathdip/) was performed to inquire the relevant signaling pathways of ARL2 in KEGG and Reactome (Additional file 4: Figure S4E). It was revealed that ARL2 was relevant to several downstream signaling pathways, including PI3K-Akt, ERK/MAPK and EGFR. These pathways were also downstream targets relevant to AXL [42, 43]. In addition, previous report and our previous study proved that AXL played a critical role in the functional regulation of glioma cells [29, 45]. Based on these observations, we further investigated the effect of ARL2 expression on AXL in glioma cells. Firstly, western blot was applied to examine AXL expression in glioma cells infected with ARL2 overexpression or control vector. The results demonstrated that ARL2 overexpression decreased AXL protein expression (Fig. 4b, *P* = 0.038, and c *P* = 0.0053). Secondly, immunocytochemistry confirmed that ARL2 overexpression attenuated AXL expression in U251 cells (Fig. 4d). Thirdly, IHC staining of ARL2 and AXL in U87 xenograft were consistent with the results of western blot and ICC (Fig. 4e). The upregulated ARL2 expression induced a reduced AXL expression. Finally, phospho-AXL (Tyr702) protein expression in U251 cells was also inhibited after ARL2 overexpression (Fig. 4f, *P* = 0.0018). We also observed that the expression level of phospho-ERK decreased in U251 cells with ARL2 overexpression (Fig. 4g, *P* < 0.01). In contrast, there was no significant change in total ERK, total AKT and phospho-AKT expression (Fig. 4f and Additional file 5: Figure S5). We further performed qPCR to detect *ARL2* and *AXL* mRNA level in U251 cells transfected with ARL2 overexpression vector or control vector. The data demonstrated that the expression level of ARL2 was increased significantly after transduction (Additional file 6: Figure S6A, *P* < 0.0001). But *ARL2* overexpression didn’t lead to significant decrease in *AXL* mRNA expression (Additional file 6: Figure S6B, *P* = 0.7087). In addition, Ubibrowser (http://ubibrowser.ncpsb.org/) were applied to analyze the high confidence E3 ligases that interacted with AXL. The result showed that STUB1 was one of high confidence E3 ligases that interacted with AXL (Additional file 6: Figure S6C). Finally, TCGA data were used to investigate whether these genes were coexpressed with ARL2. The result showed that STUB1 was positively correlated to ARL2 expression (Additional file 6: Figure S6D). Altogether, these results indicated that ARL2 overexpression suppressed the expression of AXL and the activation of ERK in glioma cells.

### AXL overexpression partially rescued the phenotype induced by ARL2 overexpression in U251 cells

**Fig. 5:**
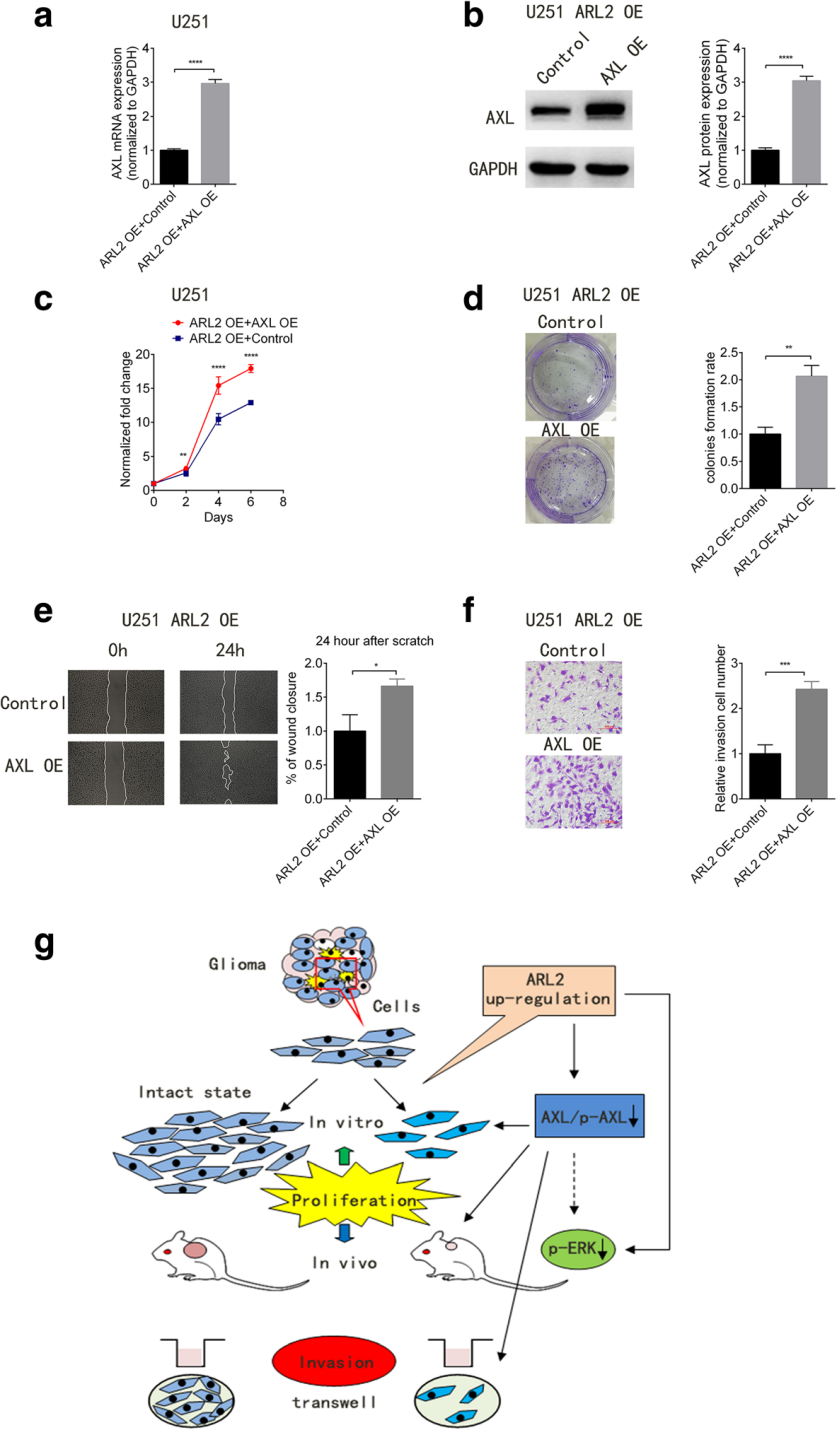
AXL overexpression partially restores the phenotype change induced by ARL2 overexpression. **a** qPCR analyses of AXL in U251 cells transduced with lentiviral ARL2 overexpression vector together with AXL overexpression or control vector. (*P* < 0.0001, *n* = 3, *t* test). **b** Western blot images and analyses of AXL in U251 glioma cells transduced with lentiviral ARL2 overexpression vector together with AXL overexpression or control vector. (*P* < 0.0001, *n* = 3, *t* test). **c** In vitro growth assay showed that the inhibition of U251 proliferation induced by ARL overexpression could be partly rescued by AXL overexpression. (Day 2,*P* = 0.0055;Day 4,*P* < 0.0001; Day 6, *P* < 0.0001; *n* = 6, *t* test). **d** Colony formation assay revealed that the inhibition of clone formation capabilities in U251 cells induced by ARL2 overexpression could be partially rescued by AXL overexpression. (1000 cells/well, *P* < 0.001, *n* = 3, *t* test). **e** Wound healing test showed that the inhibition of migration capabilities in U251 cells induced by ARL2 overexpression could be partially rescued by AXL overexpression. (*P* < 0.01, *n* = 3, *t* test). **f** Transwell assay confirmed that AXL overexpression can partially rescued the invasion capability inhibition in U251 cells induced by ARL2 overexpression. Scale bar, 100 μm. (*P* < 0.01, *n* = 3, *t* test). **g** The diagram illustrated that ARL2 up-regulation decreased the capabilities of proliferation, invasion and tumorigenesis via inhibiting the expression of AXL in glioma cells

To explore the physiological role of ARL2-AXL axis in glioma cells, we evaluated whether AXL overexpression rescue the phenotype induce by ARL2 overexpression in glioma cells. Therefore, we transduced ARL2 overexpression U251 cells with lentiviral AXL overexpression vector (Fig. 5a and b). As a result, the reduced in vitro cell growth and clone formation capabilities of U251 cells by ARL2 overexpression were partially restored by AXL overexpression (Fig. 5c and d). Consistently, we found that their inhibited migration and invasive abilities by ARL2 overexpression were also partially rescued by AXL overexpression, yet not completely (Fig. 5e and f).

## Discussion

Microtubule network dynamics is crucial to the regulation of physiological processes like cell mitosis and migration. As a key regulator of microtubule, ARL2 has been implicated in several malignant tumors, such as breast cancer, cervical cancer, and pancreatic cancer [24, 26, 46]. But the pathophysiologic role and expression pattern of ARL2 in cancer is still controversial [23, 24], and the function of ARL2 in glioma remains unknown. In this study, we identified ARL2 expression pattern and its clinical significance in glioma. The downregulation of ARL2 implies the poor prognosis in glioma patients. Furthermore, our results confirmed that ARL2 reduced the growth, clone formation, migration and invasive abilities of glioma cells, as well as in vivo tumorigenicity. These data indicate a promising potential role of ARL2 in malignant glioma treatment.

Another novel finding in this study is that AXL expression is regulated by ARL2. AXL is a member of the TAM (TYRO3, AXL, MER) subfamily of receptor tyrosine kinases [47]. Earlier report and our previous works have described the function of AXL in regulating cell growth, migration and tumorigenesis of glioma [29, 45]. Our study provides the first evidence for the role of ARL2 upregulation in modifying AXL expression. To clarify the mechanism that ARL2 reduced the expression of AXL, qPCR was performed to detect *ARL2* and *AXL* mRNA level in U251 cells after ARL2 overexpression. The result showed that *ARL2* overexpression didn’t lead to significant decrease in *AXL* mRNA expression. Therefore, we concluded that ARL2 might regulate AXL expression through post-transcriptional mechanism. Previous studies showed that Ras family members like Rab35, Rac1, Cdc42 and Rnd3 mediate ubiquitin modification [48–50]. Based on these observations, we used Ubibrowser to analyze the high confidence E3 ligases that interacted with AXL. We also applied TCGA data to explore whether these genes were coexpressed with ARL2. The result showed that STUB1 was not only one of high confidence E3 ligases that interacted with AXL, but also positively correlated to ARL2 expression. Taken together, these results indicate that ubiquitination and degradation may be a possible mechanism how ARL2 regulate AXL expression. Further studies are needed to fully elucidate the detail mechanism of ARL2-AXL axis. In addition, the restoration of AXL by exogenous expression did not fully rescue the defects in U251 glioma cells caused by ARL2 overexpression, which suggested that there might be additional molecular downstream targets associated with ARL2 overexpression.

## Conclusion

In conclusion, this study described the downregulation of ARL2 in clinical glioma samples and its clinical relevance to poor prognosis in glioma patients. Secondly, this study provided the evidence that elevated ARL2 expression in glioma cell lines inhibits the abilities of proliferation, clone formation, migration and invasion. Thirdly, we demonstrated that ARL2 was associated with the regulation of tumorigenicity of glioma cells in vivo. Finally, it was proved in this study that ARL2 regulated AXL expression and activated phospho-ERK in glioma. Altogether, our data suggest that ARL2 serves as an important suppressor for the proliferation, migration and tumorigenicity of glioma cells by regulating the expression of AXL. Therefore, it may conduct as a new prognostic and therapeutic target for glioma. Supplementary methods are available in Additional file 7.

## Additional files

Additional file 1: **Figure S1.** The mRNA expression level of ARL family members in GBM and non-tumor samples from TCGA. (TIF 266 kb)

Additional file 2: **Figure S2.** ARL2 expression decreased in GBM. (TIF 459 kb)

Additional file 3: **Figure S3.** ARL2 overexpression increased the proportion of cells at G0/G1 phase and decreased the proportion of cells at S and G2/M phase. (TIF 416 kb)

Additional file 4: **Figure S4.** The relevant signaling pathway analysis showed that ARL2 expression was correlated with EGFR and AXL signaling. (TIF 1460 kb)

Additional file 5: **Figure S5.** Western blot showed that ARL2 overexpression in U251 cells didn’t affect the expression of total and phospho-form AKT. (TIF 842 kb)

Additional file 6: **Figure S6.** ARL2 overexpression didn’t increase the expression of AXL mRNA. (TIF 674 kb)

Additional file 7: Supplementary Methods. (DOCX 17 kb)

## Abbreviations

ARF: ADP-ribosylation factor
ARL2: ADP ribosylation factor-like GTPase 2
BART: Binder of ADP-ribosylation factor-like two
CGGA: Chinese Glioma Genome Atlas
FPS: median progression-free survival
GSEA: Gene Set Enrichment Analysis
PP2A: Protein phosphatase 2A
STUB1: E3 ubiquitin-protein ligase CHIP
TCGA: The Cancer Genome Atlas

## References

[1] Wen PY, Kesari S (2008). Malignant gliomas in adults. N Engl J Med.

[2] Prados MD, Yung WK, Wen PY, Junck L, Cloughesy T, Fink K, Chang S, Robins HI, Dancey J, Kuhn J (2008). Phase-1 trial of gefitinib and temozolomide in patients with malignant glioma: a north American brain tumor consortium study. Cancer Chemother Pharmacol.

[3] Fine HA (2015). New strategies in glioblastoma: exploiting the new biology. Clin Cancer Res.

[4] Matozaki T, Nakanishi H, Takai Y (2000). Small G-Protein networks: their crosstalk and signal cascades. Cell Signal.

[5] Paduch M, Jelen F, Otlewski J (2001). Structure of small G proteins and their regulators. Acta Biochim Pol.

[6] Zhang F, Cheong JK (2016). The renewed battle against RAS-mutant cancers. Cellular and molecular life sciences : CMLS.

[7] Stephen AG, Esposito D, Bagni RK, McCormick F (2014). Dragging ras back in the ring. Cancer Cell.

[8] Cox AD, Fesik SW, Kimmelman AC, Luo J, Der CJ (2014). Drugging the undruggable RAS: mission possible?. Nat Rev Drug Discov.

[9] Clark J, Moore L, Krasinskas A, Way J, Battey J, Tamkun J, Kahn RA (1993). Selective amplification of additional members of the ADP-ribosylation factor (ARF) family: cloning of additional human and Drosophila ARF-like genes. Proc Natl Acad Sci U S A.

[10] Bhamidipati A, Lewis SA, Cowan NJ (2000). ADP ribosylation factor-like protein 2 (Arl2) regulates the interaction of tubulin-folding cofactor D with native tubulin. J Cell Biol.

[11] Newman LE, Zhou CJ, Mudigonda S, Mattheyses AL, Paradies E, Marobbio CM, Kahn RA (2014). The ARL2 GTPase is required for mitochondrial morphology, motility, and maintenance of ATP levels. PLoS One.

[12] Chen K, Koe CT, Xing ZB, Tian X, Rossi F, Wang C, Tang Q, Zong W, Hong WJ, Taneja R (2016). Arl2- and Msps-dependent microtubule growth governs asymmetric division. J Cell Biol.

[13] Francis JW, Newman LE, Cunningham LA, Kahn RA, Trimer A (2017). Consisting of the tubulin-specific chaperone D (TBCD), regulatory GTPase ARL2, and beta-tubulin is required for maintaining the microtubule network. J Biol Chem.

[14] Nithianantham S, Le S, Seto E, Jia W, Leary J, Corbett KD, Moore JK, Al-Bassam J. Tubulin cofactors and Arl2 are cage-like chaperones that regulate the soluble alphabeta-tubulin pool for microtubule dynamics. eLife. 2015;4:e08811. 10.7554/eLife.08811 PMC4574351 26208336

[15] Zhou C, Cunningham L, Marcus AI, Li Y, Kahn RA (2006). Arl2 and Arl3 regulate different microtubule-dependent processes. Mol Biol Cell.

[16] Newman LE, Schiavon CR, Zhou C, Kahn RA (2017). The abundance of the ARL2 GTPase and its GAP, ELMOD2, at mitochondria are modulated by the fusogenic activity of mitofusins and stressors. PLoS One.

[17] Ismail SA, Chen YX, Rusinova A, Chandra A, Bierbaum M, Gremer L, Triola G, Waldmann H, Bastiaens PI, Wittinghofer A (2011). Arl2-GTP and Arl3-GTP regulate a GDI-like transport system for farnesylated cargo. Nat Chem Biol.

[18] Muromoto R, Sekine Y, Imoto S, Ikeda O, Okayama T, Sato N, Matsuda T (2008). BART is essential for nuclear retention of STAT3. Int Immunol.

[19] Zhang T, Li S, Zhang Y, Zhong C, Lai Z, Ding J (2009). Crystal structure of the ARL2-GTP-BART complex reveals a novel recognition and binding mode of small GTPase with effector. Structure.

[20] Bailey LK, Campbell LJ, Evetts KA, Littlefield K, Rajendra E, Nietlispach D, Owen D, Mott HR (2009). The structure of binder of Arl2 (BART) reveals a novel G protein binding domain: implications for function. J Biol Chem.

[21] Zhou Y, Jiang H, Gu J, Tang Y, Shen N, Jin Y (2013). MicroRNA-195 targets ADP-ribosylation factor-like protein 2 to induce apoptosis in human embryonic stem cell-derived neural progenitor cells. Cell Death Dis.

[22] Beghin A, Honore S, Messana C, Matera EL, Aim J, Burlinchon S, Braguer D, Dumontet C (2007). ADP ribosylation factor like 2 (Arl2) protein influences microtubule dynamics in breast cancer cells. Exp Cell Res.

[23] Hass HG, Vogel U, Scheurlen M, Jobst J (2016). Gene-expression analysis identifies specific patterns of dysregulated molecular pathways and genetic subgroups of human hepatocellular carcinoma. Anticancer Res.

[24] Peng R, Men J, Ma R, Wang Q, Wang Y, Sun Y, Ren J (2017). miR-214 down-regulates ARL2 and suppresses growth and invasion of cervical cancer cells. Biochem Biophys Res Commun.

[25] Beghin A, Belin S, Hage-Sleiman R, Brunet Manquat S, Goddard S, Tabone E, Jordheim LP, Treilleux I, Poupon MF, Diaz JJ (2009). ADP ribosylation factor like 2 (Arl2) regulates breast tumor aggressivity in immunodeficient mice. PLoS One.

[26] Beghin A, Matera EL, Brunet-Manquat S, Dumontet C (2008). Expression of Arl2 is associated with p53 localization and chemosensitivity in a breast cancer cell line. Cell Cycle.

[27] Wang K, Li P, Dong Y, Cai X, Hou D, Guo J, Yin Y, Zhang Y, Li J, Liang H (2011). A microarray-based approach identifies ADP ribosylation factor-like protein 2 as a target of microRNA-16. J Biol Chem.

[28] Gioia R, Leroy C, Drullion C, Lagarde V, Etienne G, Dulucq S, Lippert E, Roche S, Mahon FX, Pasquet JM (2011). Quantitative phosphoproteomics revealed interplay between Syk and Lyn in the resistance to nilotinib in chronic myeloid leukemia cells. Blood.

[29] Cheng P, Phillips E, Kim SH, Taylor D, Hielscher T, Puccio L, Hjelmeland AB, Lichter P, Nakano I, Goidts V (2015). Kinome-wide shRNA screen identifies the receptor tyrosine kinase AXL as a key regulator for mesenchymal glioblastoma stem-like cells. Stem cell reports.

[30] Cheng P, Wang J, Waghmare I, Sartini S, Coviello V, Zhang Z, Kim SH, Mohyeldin A, Pavlyukov MS, Minata M (2016). FOXD1-ALDH1A3 signaling is a determinant for the self-renewal and Tumorigenicity of mesenchymal glioma stem cells. Cancer Res.

[31] Sun L, Hui AM, Su Q, Vortmeyer A, Kotliarov Y, Pastorino S, Passaniti A, Menon J, Walling J, Bailey R (2006). Neuronal and glioma-derived stem cell factor induces angiogenesis within the brain. Cancer Cell.

[32] Griesinger AM, Birks DK, Donson AM, Amani V, Hoffman LM, Waziri A, Wang M, Handler MH, Foreman NK (2013). Characterization of distinct immunophenotypes across pediatric brain tumor types. J Immunol.

[33] Cheng W, Zhang C, Ren X, Jiang Y, Han S, Liu Y, Cai J, Li M, Wang K, Liu Y (2017). Bioinformatic analyses reveal a distinct notch activation induced by STAT3 phosphorylation in the mesenchymal subtype of glioblastoma. J Neurosurg.

[34] Subramanian A, Tamayo P, Mootha VK, Mukherjee S, Ebert BL, Gillette MA, Paulovich A, Pomeroy SL, Golub TR, Lander ES (2005). Gene set enrichment analysis: a knowledge-based approach for interpreting genome-wide expression profiles. Proc Natl Acad Sci U S A.

[35] Cheng W, Li M, Jiang Y, Zhang C, Cai J, Wang K, Wu A (2016). Association between small heat shock protein B11 and the prognostic value of MGMT promoter methylation in patients with high-grade glioma. J Neurosurg.

[36] Jiang T, Mao Y, Ma W, Mao Q, You Y, Yang X, Jiang C, Kang C, Li X, Chen L (2016). CGCG clinical practice guidelines for the management of adult diffuse gliomas. Cancer Lett.

[37] Cormier N, Yeo A, Fiorentino E, Paxson J (2015). Optimization of the wound scratch assay to detect changes in murine mesenchymal stromal cell migration after damage by soluble cigarette smoke extract. J Vis Exp.

[38] Chen J, Tang J, Chen W, Gao Y, He Y, Zhang Q, Ran Q, Cao F, Yao S (2017). Effects of syndecan-1 on the expression of syntenin and the migration of U251 glioma cells. Oncol Lett.

[39] Wu WS, Chien CC, Liu KH, Chen YC, Chiu WT (2017). Evodiamine prevents glioma growth, induces glioblastoma cell apoptosis and cell cycle arrest through JNK activation. Am J Chin Med.

[40] Xu X, Cai N, Zhi T, Bao Z, Wang D, Liu Y, Jiang K, Fan L, Ji J, Liu N (2017). MicroRNA-1179 inhibits glioblastoma cell proliferation and cell cycle progression via directly targeting E2F transcription factor 5. Am J Cancer Res.

[41] Naito S, von Eschenbach AC, Giavazzi R, Fidler IJ (1986). Growth and metastasis of tumor cells isolated from a human renal cell carcinoma implanted into different organs of nude mice. Cancer Res.

[42] Guo G, Gong K, Ali S, Ali N, Shallwani S, Hatanpaa KJ, Pan E, Mickey B, Burma S, Wang DH (2017). A TNF-JNK-Axl-ERK signaling axis mediates primary resistance to EGFR inhibition in glioblastoma. Nat Neurosci.

[43] Zhang Z, Lee JC, Lin L, Olivas V, Au V, LaFramboise T, Abdel-Rahman M, Wang X, Levine AD, Rho JK (2012). Activation of the AXL kinase causes resistance to EGFR-targeted therapy in lung cancer. Nat Genet.

[44] Brand TM, Iida M, Corrigan KL, Braverman CM, Coan JP, Flanigan BG, Stein AP, Salgia R, Rolff J, Kimple RJ, et al. The receptor tyrosine kinase AXL mediates nuclear translocation of the epidermal growth factor receptor. Sci Signal. 2017;10(460):eaag1064. 10.1126/scisignal.aag1064 PMC7094775 28049763

[45] Vajkoczy P, Knyazev P, Kunkel A, Capelle HH, Behrndt S, von Tengg-Kobligk H, Kiessling F, Eichelsbacher U, Essig M, Read TA (2006). Dominant-negative inhibition of the Axl receptor tyrosine kinase suppresses brain tumor cell growth and invasion and prolongs survival. Proc Natl Acad Sci U S A.

[46] Taniuchi K, Nishimori I, Hollingsworth MA (2011). Intracellular CD24 inhibits cell invasion by posttranscriptional regulation of BART through interaction with G3BP. Cancer Res.

[47] O'Bryan JP, Frye RA, Cogswell PC, Neubauer A, Kitch B, Prokop C, Espinosa R, Le Beau MM, Earp HS, Liu ET (1991). Axl, a transforming gene isolated from primary human myeloid leukemia cells, encodes a novel receptor tyrosine kinase. Mol Cell Biol.

[48] Minowa-Nozawa A, Nozawa T, Okamoto-Furuta K, Kohda H, Nakagawa I (2017). Rab35 GTPase recruits NPD52 to autophagy targets. EMBO J.

[49] Ma J, Xue Y, Liu W, Yue C, Bi F, Xu J, Zhang J, Li Y, Zhong C, Chen Y (2013). Role of activated Rac1/Cdc42 in mediating endothelial cell proliferation and tumor angiogenesis in breast cancer. PLoS One.

[50] Liu B, Dong H, Lin X, Yang X, Yue X, Yang J, Li Y, Wu L, Zhu X, Zhang S (2016). RND3 promotes snail 1 protein degradation and inhibits glioblastoma cell migration and invasion. Oncotarget.

