# Supplementary figures and images for: ARL2 overexpression inhibits glioma proliferation and tumorigenicity via down-regulating AXL
Source: BMC Cancer. 2018 May 29;18:599. doi: 10.1186/s12885-018-4517-0 (PMC5975491; doi:10.1186/s12885-018-4517-0)

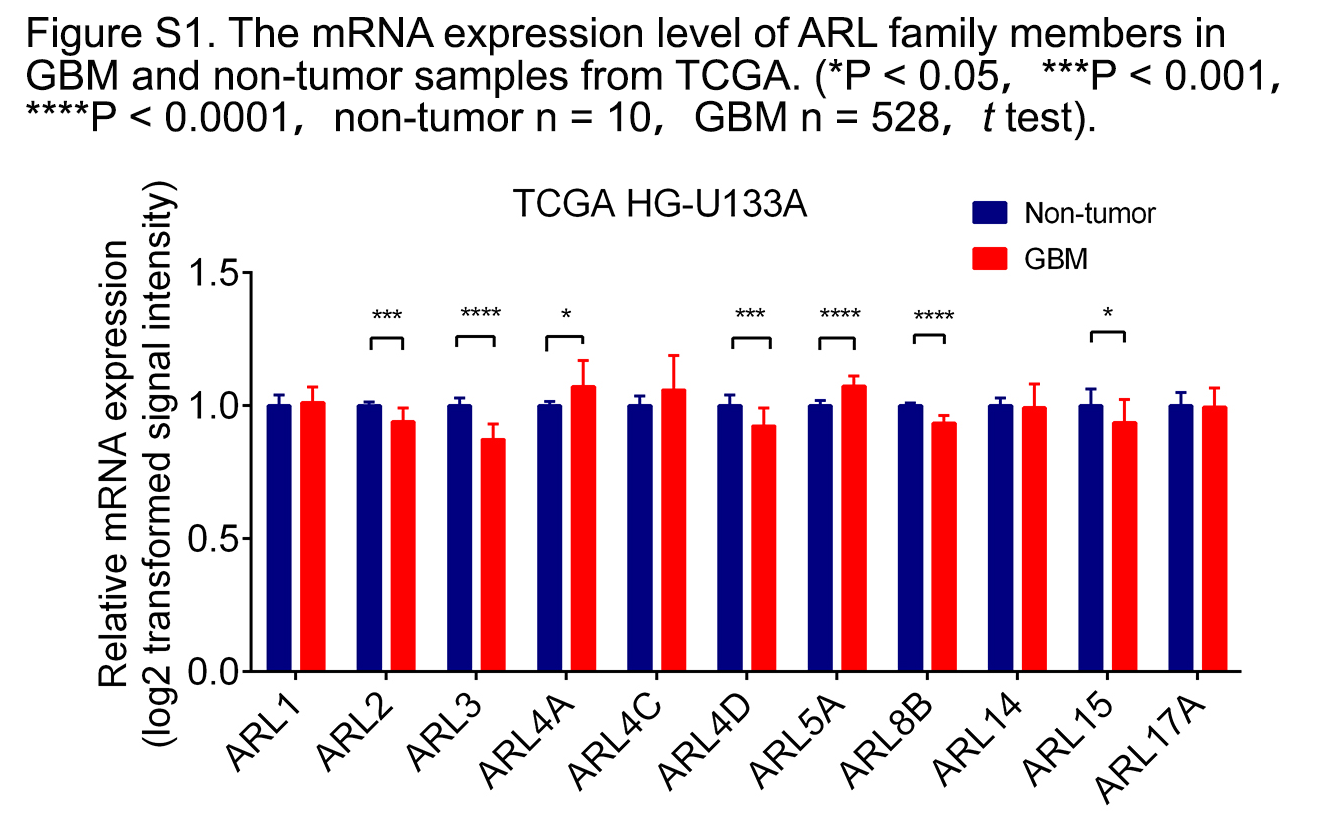

Supplement: Supplementary file 1 — Figure S1. The mRNA expression level of ARL family members in GBM and non-tumor samples from TCGA. (TIF 266 kb) [file 12885_2018_4517_MOESM1_ESM.tif]

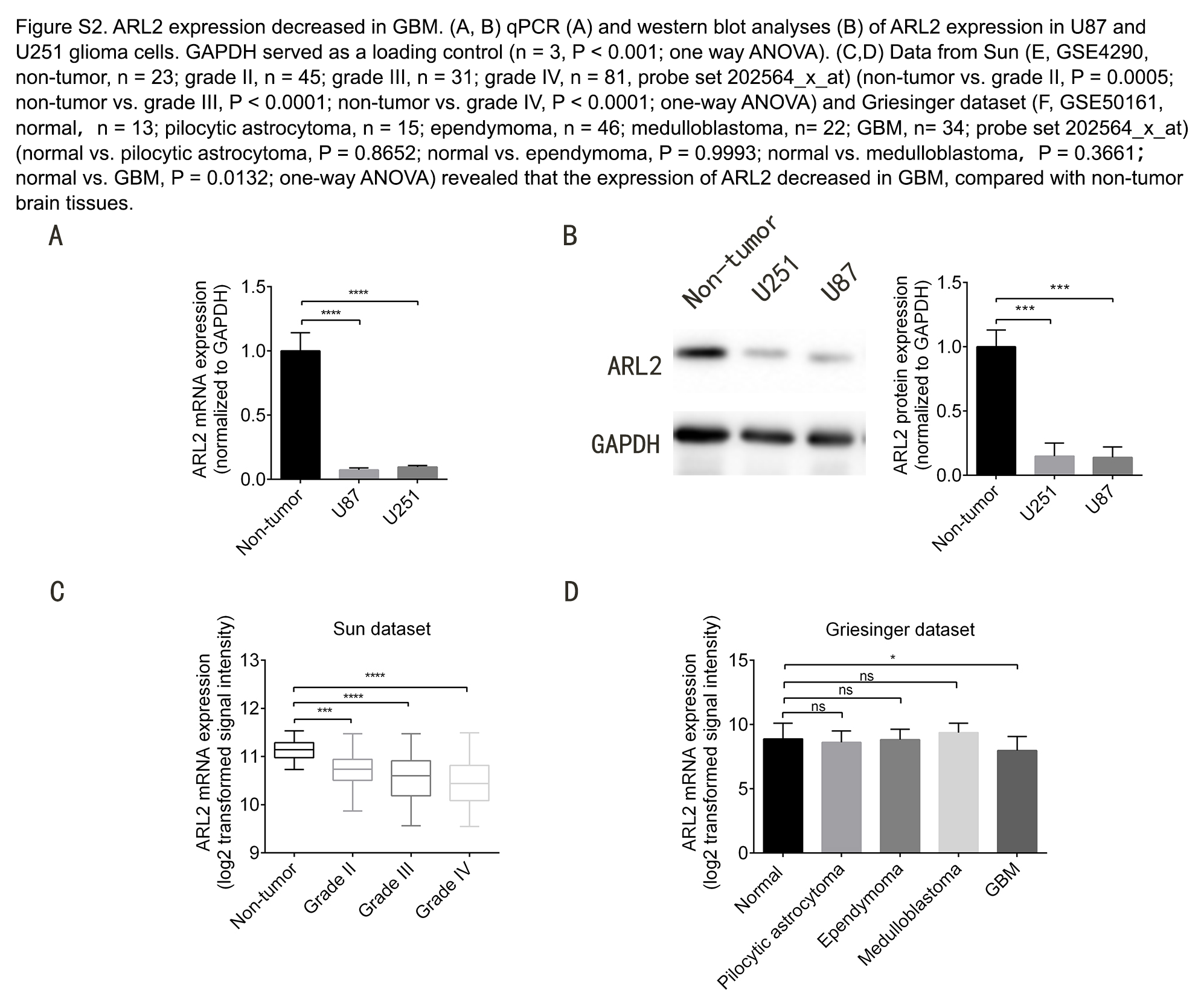

Supplement: Supplementary file 2 — Figure S2. ARL2 expression decreased in GBM. (TIF 459 kb) [file 12885_2018_4517_MOESM2_ESM.tif]

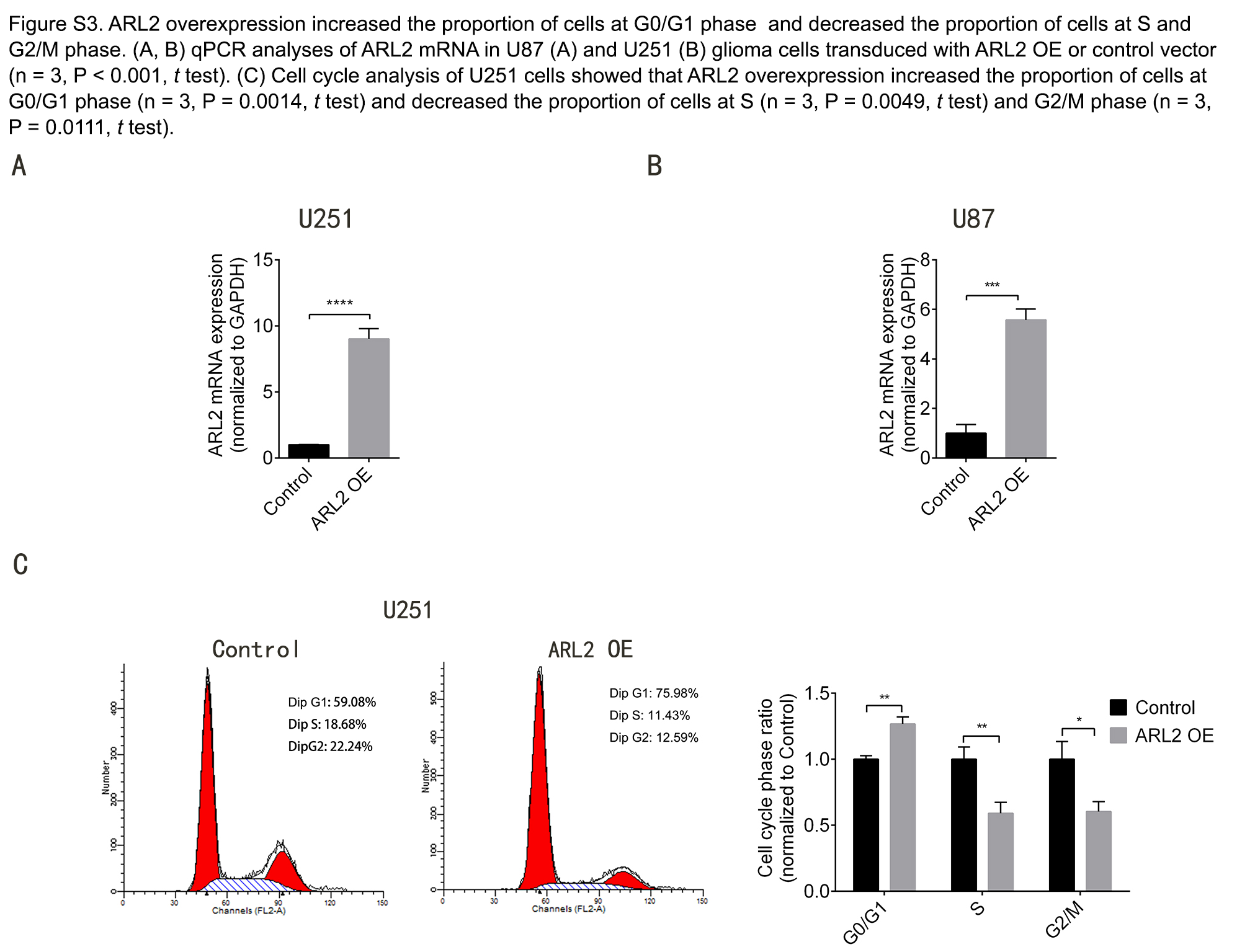

Supplement: Supplementary file 3 — Figure S3. ARL2 overexpression increased the proportion of cells at G0/G1 phase and decreased the proportion of cells at S and G2/M phase. (TIF 416 kb) [file 12885_2018_4517_MOESM3_ESM.tif]

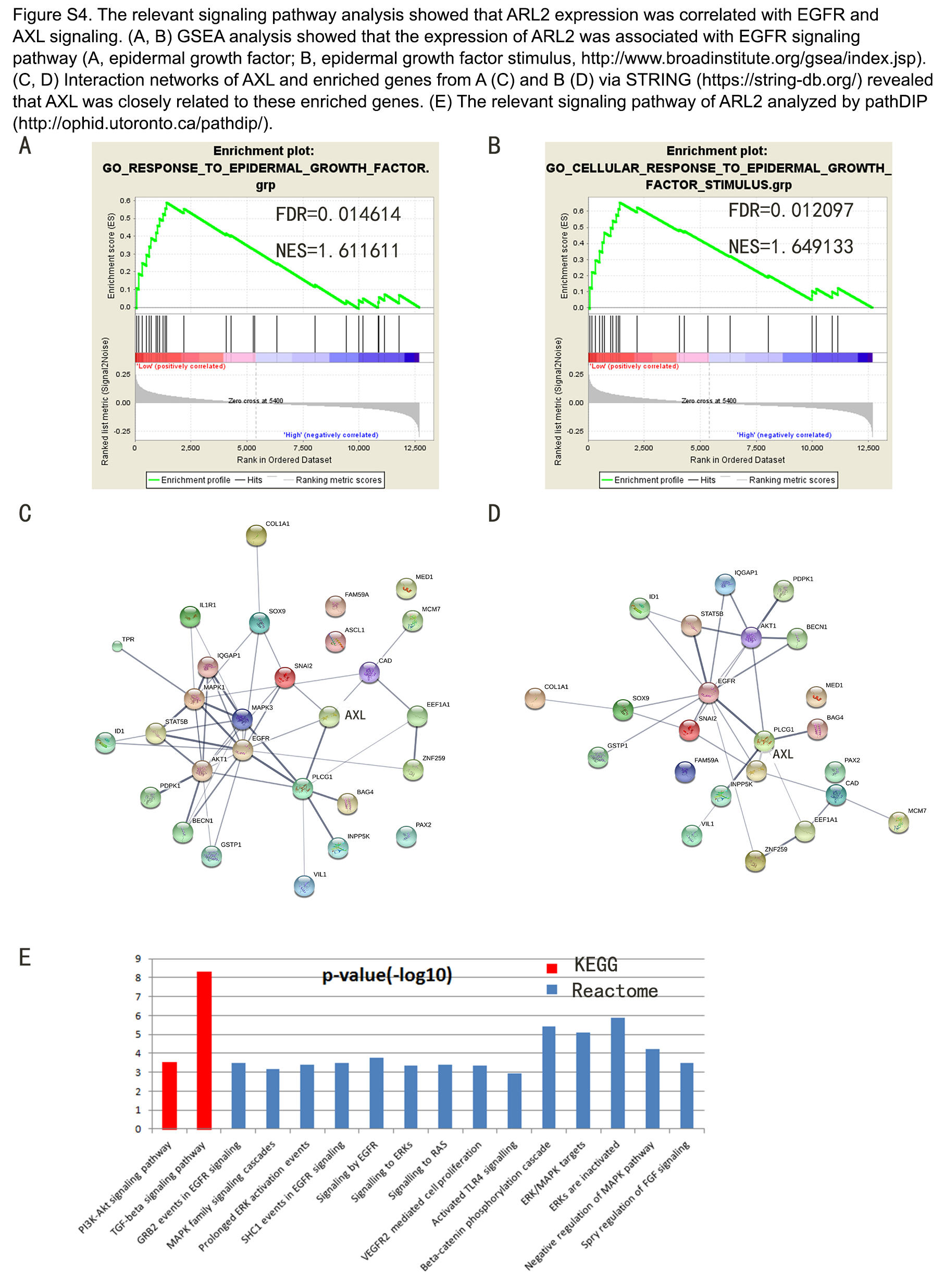

Supplement: Supplementary file 4 — Figure S4. The relevant signaling pathway analysis showed that ARL2 expression was correlated with EGFR and AXL signaling. (TIF 1460 kb) [file 12885_2018_4517_MOESM4_ESM.tif]

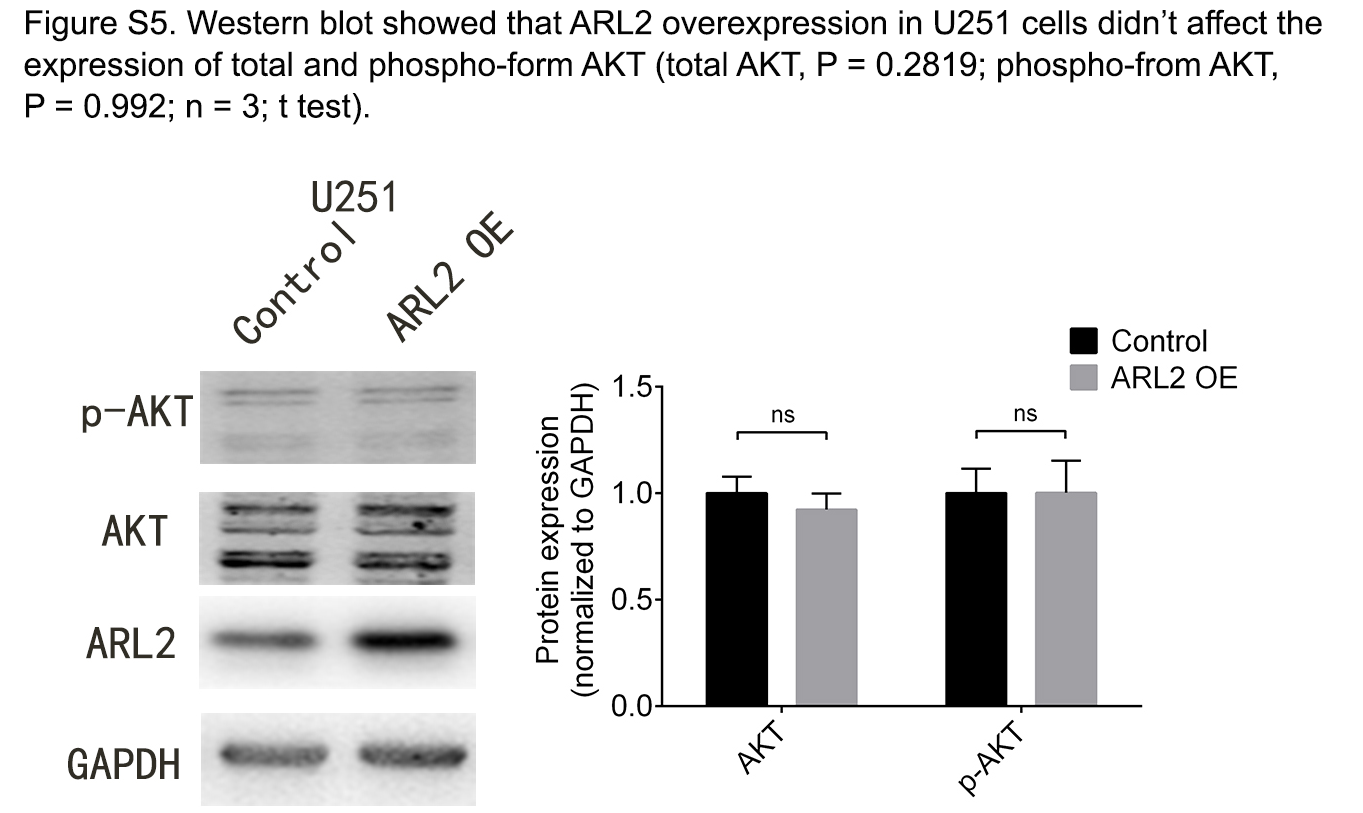

Supplement: Supplementary file 5 — Figure S5. Western blot showed that ARL2 overexpression in U251 cells didn’t affect the expression of total and phospho-form AKT. (TIF 842 kb) [file 12885_2018_4517_MOESM5_ESM.tif]

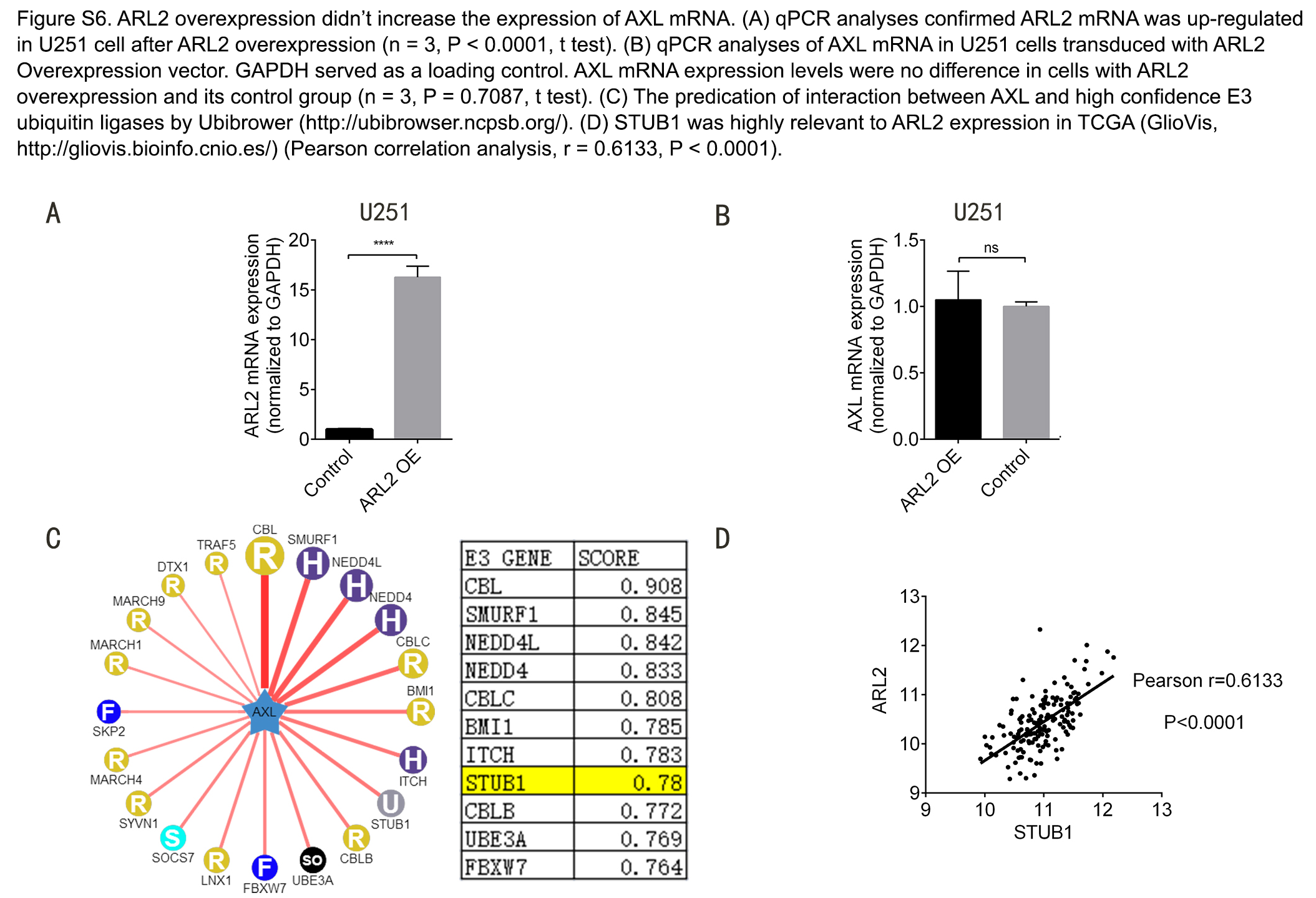

Supplement: Supplementary file 6 — Figure S6. ARL2 overexpression didn’t increase the expression of AXL mRNA. (TIF 674 kb) [file 12885_2018_4517_MOESM6_ESM.tif]
