# Supplementary material for: ARL2 overexpression inhibits glioma proliferation and tumorigenicity via down-regulating AXL
Source: BMC Cancer. 2018 May 29;18:599. doi: 10.1186/s12885-018-4517-0 (PMC5975491; doi:10.1186/s12885-018-4517-0)
Supplement: Supplementary file 7 — Supplementary Methods. (DOCX 17 kb) [file 12885_2018_4517_MOESM7_ESM.docx]

**Supplementary Methods:**

**Reagents and Antibodies**

The following reagents and antibodies were used in this study: The primary antibodies used in this study were as follows: ARL2 (Abcam, ab183510) for western blot and immunohistochemistry; GAPDH (Proteintech, 10491-1-AP), anti-ERK 1/2 (Phospho-Thr202/Tyr204) (Abcam, ab214362), ERK (Proteintech, 66192-1-Ig), phospho-Akt (Ser473) (Cell signaling technology, 9271), AKT (Cell signaling technology, 4691), phospho-AXL (Tyr702) (Cell signaling technology, 5724), and AXL (Cell signaling technology, 8661), peroxidase-conjugated Affinipure goat anti-rabbit and anti-mouse IgG (H+L) (Proteintech, SA00001-2) for western blot. Polybrene (Millipore, TR-1003-G), CCK8 (DojinDo, CK04), RIPA buffer (Sigma, R0278), whole cell lysis kit (Wanleibio, WLA019b), BCA protein assay kit (Beyotime, P0012), phophatase inhibitor cocktail (Sigma, P0044), protease inhibitor cocktail (Sigma, P8340), and western blot kit (Transgen Biotech, DW101-02). Cell culture: high-glucose DMEM (Hyclone, SH30256.01), fetal bovine serum (Serapro, S601P-500), phosphate buffered saline (1×) (SH3025601), penicillin-streptomycin solution, SV30010). PCR: TRIzol reagent (Invitrogen, 15596-026), *TransStart* top green qPCR supermix (Transgen Biotech, AQ131), and *TransScript* all-in-one first-strand cDNA synthesis supermix for qPCR (Transgen Biotech, AT341). Immunocytochemistry: DAPI (Boster, AR1176), anti-fade solution (Boster, AR1109), DAB Kit (ZSGB-BIO, ZLI-9018), SPlink detection systems (ZSGB-BIO, SP9001), and rhodamine (TRITC)-conjugated Affinipure goat anti-rabbit IgG(H+L) (Proteintech, SA00007-2). Transwell: Matrigel basement membrane matrix (Corning, 356234); crystal violet staining solution (Beyotime, C0121); cell cycle: cell cycle detection kit (KeyGEN BioTECH, KGA512).

**Cell cycle analysis**

Cells were harvested and washed with precooled PBS. 1ml cell suspension containing 10^6^ cells was fixed with 70% ice-cold ethanol 500μl overnight at 4°C, followed by incubation with RNase A 100μl (KeyGEN) at 37°C for 30 mins. Then 400μl of propidium iodide (KeyGEN) was added into the cell suspension After incubation shielded from light at 4°C for 30 mins, the samples were analyzed by fluorescence activated cell sorting (FACS, Becton-Dickinson).
